# Crystal structure of 5-butyl­amino-3-methyl-1-(pyridin-2-yl)-1*H*-pyrazole-4-carbaldehyde obtained from a microwave-assisted reaction using caesium carbonate as catalyst

**DOI:** 10.1107/S2056989016017187

**Published:** 2016-10-28

**Authors:** Mario A. Macías, Jessica Orrego-Hernández, Jaime Portilla

**Affiliations:** a Department of Chemistry, Universidad de los Andes, Carrera 1 No. 18A 10, Bogotá, Colombia; b Bioorganic Compounds Research Group, Department of Chemistry, Universidad de los Andes, Carrera 1 No. 18A 10, Bogotá, Colombia

**Keywords:** crystal structure, pharmaceutical compound, 5-amino­pyrazoles, nucleophilic substitution, hydrogen bonding

## Abstract

The title compound, C_14_H_18_N_4_O, synthesized from an unconventional microwave-assisted method using caesium carbonate as catalyst, has an approximately planar conformation with the pyridyl and pyrazole rings inclined by a dihedral angle of 7.94 (3)°, allowing the formation of an intra­molecular N—H⋯N hydrogen bond. The supra­molecular assembly has a three-dimensional arrangement controlled mainly by weak C—H⋯O and C—H⋯π inter­actions.

## Abstract 2

The new compound 5-butyl­amino-3-methyl-1-(pyridin-2-yl)-1*H*-pyrazole-4-carbaldehyde has been synthesized using a microwave-assisted reaction.

## Chemical context   

Pyrazole derivatives are compounds with notable biological activity (Peng *et al.*, 2013 ▸) and some derivatives have the capacity to form complexes with metal ions (Budzisz *et al.*, 2009 ▸). Currently, 5-amino­pyrazoles have been found to play an important role as biologically active compounds (Zhang *et al.*, 2014 ▸). As such, they are considered to be building blocks of high inter­est for pharmaceutical agents (Sakya *et al.*, 2006 ▸) and agrochemicals (Yuan *et al.*, 2013 ▸). Recently, our research group reported the chemoselective synthesis of 5-alkyl­amino-1*H*-pyrazole-4-carbaldehydes in which C—N bond formation in pyrazole rings were efficiently assisted by using caesium carbonate under microwave irradiation with short reaction times and excellent yields (Orrego-Hernández *et al.*, 2015*a* ▸). Herein, we report the crystal structure of the new 5-(butylamino)-3-methyl-1-(pyridin-2-yl)-1*H*-pyrazole-4-carbaldehyde derived from 5-chloro-3-methyl-1-(pyridin-2-yl)-1*H*-pyrazole-4-carbaldehyde and butyl­amine by using the ‘caesium effect’ and microwave irradiation.
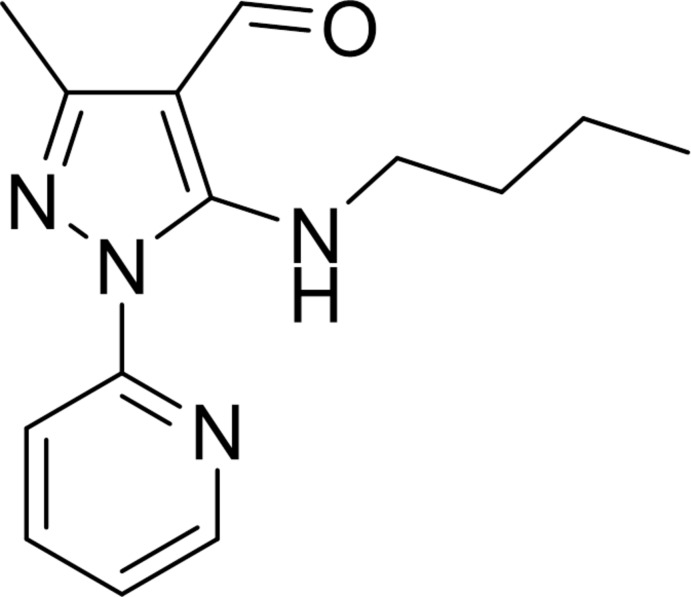


## Structural commentary   

**Figure 1:**
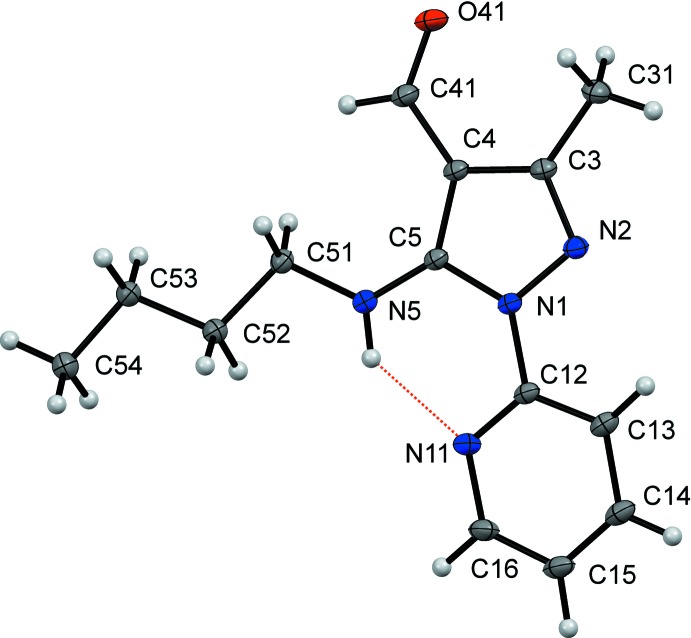
The mol­ecular structure of the title compound, showing anisotropic displacement ellipsoids drawn at the 50% probability level. The intramolecular N—H⋯N hydrogen bond is shown as a dashed line (see Table 1 ▸).

In the mol­ecular structure of the title compound (Fig. 1 ▸), the pyridyl and pyrazole rings are nearly coplanar with a dihedral angle between their planes of 7.94 (3)°. The pyridyl ring has an orientation that allows the formation of an intra­molecular N5—H1⋯N11 hydrogen bond (Fig. 1 ▸ and Table 1 ▸) to generate an *S*(6) motif. This structural feature is also observed in its analog 5-cyclo­hexyl­amino-3-methyl-1-(pyridin-2-yl)-1*H*-pyrazole-4-carbaldehyde, which even shows a smaller dihedral angle between the pyridyl and pyrazole rings [2.47 (5)°; Orrego-Hernández *et al.*, 2015*b* ▸). In both mol­ecules, the 3-methyl-1-(pyridin-2-yl)-1*H*-pyrazole-4-carbaldehyde nucleus presents a similar, but not identical, conformation with a maximum r.m.s. deviation of 0.0906 Å, keeping the atomic distances very similar in the pyrazole ring.

## Supra­molecular features   

**Figure 2:**
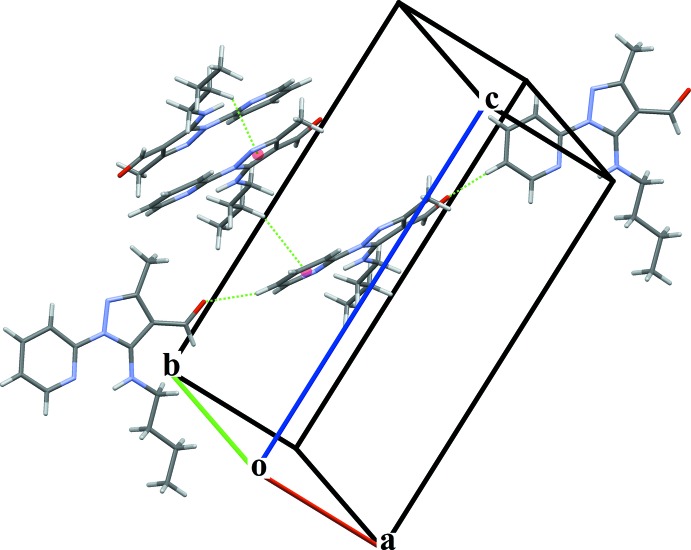
The crystal structure of the title compound, showing the C—H⋯O and C—H⋯π hydrogen-bond inter­actions.

In the crystal structure, C15—H15⋯O41^i^ [symmetry code: (i) *x* − 1, −*y* + 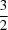, *z* − 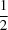] inter­actions link the mol­ecules into *C*(10) chains running along [201], see Fig. 2 ▸. Parallel chains are connected by weak C52—H52*B*⋯*Cg*1^ii^ [*Cg*1 is the centroid of the C3–C5/N1/N2 ring; symmetry code: (ii) −*x* + 1, −*y* + 1, −*z* + 2] and C53—H53*A*⋯*Cg*2^iii^ [*Cg*2 is the centroid of the N11/C12–C16 ring; symmetry code: (iii) *x* + 1, *y*, *z*] inter­actions, which help to define a three-dimensional array.

## Database survey   

A search of the Cambridge Structural Database (CSD Version 5.37 with two updates; Groom *et al.*, 2016 ▸) for the 1-(pyridin-2-yl)-1*H*-pyrazole nucleus with the possibility of any group bonded to C3, C4 or C5 gave 12 hits of which 10 correspond to organometallic compounds, one to 2-(3,5-*bis*(4-(*n*-oct­yloxy)phen­yl)pyrazol-1-yl)pyridine and the last to 2,6-*bis*(pyrazol­yl)pyridine. Any other search considering the presence of the butyl­amino or carbaldehyde groups gave no hits. However, two related compounds 5-cyclo­hexyl­amino-3-methyl-1-(pyridin-2-yl)-1*H*-pyrazole-4-carbaldehyde and (*Z*)-4-[(cyclo­hexyl­amino)­methyl­idene]-3-methyl-1-phenyl-1*H*-pyrazol-5(4*H*)-one have been published recently (Orrego-Hernández *et al.*, 2015*b* ▸). These compounds are pyrazole derivatives which, despite the overall similarities of the mol­ecular geometries and the potentially available donors and acceptors for hydrogen-bonding inter­actions, present different supra­molecular assemblies.

## Synthesis and crystallization   

**Figure 3:**
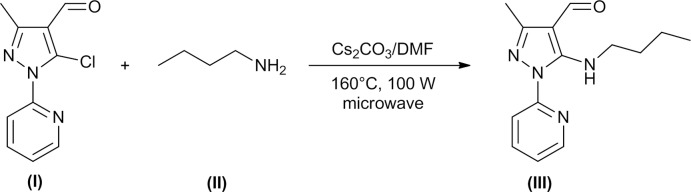
Schematic representation of the microwave-assisted reaction using caesium carbonate as catalyst.

All reactive and solvents, including caesium carbonate (99%, Aldrich), were purchased from commercial sources and used as received. A mixture of 5-chloro-3-methyl-1-(pyridin-2-yl)-1*H*-pyrazole-4-carbaldehyde [(I) in Fig. 3 ▸; 0.100 g, 0.45 mmol, 1 equiv.], butyl­amine [(II) in Fig. 3 ▸; 0.56 mmol, 1.3 equiv.], caesium carbonate (0.029 g, 20% mmol, 0.2 equiv.) and 2 mL of di­methyl­formamide (DMF) were placed in a reaction tube of a CEM DiscoverTM, containing a magnetic stirring bar. The tube was sealed with a plastic microwave septum and was irradiated at 433 K for 25 min at 100 W. The resulting crude product was partitioned between di­chloro­methane and water. The organic layer was washed with water, then brine, and dried over anhydrous sodium sulfate. Subsequently, the solvent was removed under vacuum and the residue was purified by silica gel flash chromatography (DCM) to afford 5-(butyl­amino)-3-methyl-1-(pyridin-2-yl)-1*H*-pyrazole-4-carb­aldehyde [(III) in Fig. 3 ▸]. Yellow crystals of (III) suitable for single-crystal X-ray diffraction were grown in DMF by slow evaporation, at ambient temperature and in air, [94% yield, m.p. 354 K]. HRMS (ESI+): [*M* + H]^+^ calculated for C_14_H_19_N_4_O^+^ 259.1553, found 259.1546. Yield 0.109 g, 94%; m.p. 348–350 K; IR ν_max_ (KBr): 3448, 3211, 3096, 2924, 2858, 1643, 1596, 1563, 1436, 1002 cm^−1^; ^1^H NMR (CDCl_3_): 0.95 (*t*, *J* = 7.4, 3H), 1.44 (*m*, 2H), 1.68 (*m*, 2H), 2.44 (*s*, 3H), 3.60 (*t*, *J* = 7.1 Hz, 2H), 7.10 (*t*, *J* = 5.2 Hz, 1H), 7.78 (*t*, *J* = 7.0 Hz, 1H), 7.93 (*d*, *J* = 8.4 Hz, 1H), 8.28 (*d*, *J* = 4.8 Hz, 1H), 9.82 (*s*, 1H); ^13^C NMR (CDCl_3_): 13.7 (CH_3_), 14.5 (CH_3_), 19.9 (CH_2_), 32.0 (CH_2_), 46.4 (CH_2_), 106.6 (C), 114.0 (CH), 119.8 (CH), 138.8 (CH), 145.8 (CH), 152.8 (C), 153.0 (C), 154.3 (C), 182.0 (CH); MS (EI) *m*/*z* 258 (*M* ^+^, 26%), 215 (67), 187 (59), 134 (32), 93 (47), 78 (76), 51 (24), 32 (100); HRMS *m*/*z* (ESI) calculated for [C_14_H_18_N_4_O+H]^+^: 259.1553; found 259.1546 [(*M* + H)^+^].

## Refinement   

Crystal data, data collection and structure refinement details are summarized in Table 2 ▸. H atoms were placed in calculated positions (C—H = 0.95–0.99 Å) and included as riding with isotropic displacement parameters set at 1.2–1.5 times the *U*_eq_ value of the parent atom. H atoms belonging to NH groups were located in difference density maps and were freely refined.

## Supplementary Material

Crystal structure: contains datablock(s) I. DOI: 10.1107/S2056989016017187/bg2597sup1.cif

Structure factors: contains datablock(s) I. DOI: 10.1107/S2056989016017187/bg2597Isup2.hkl

Supporting information file. DOI: 10.1107/S2056989016017187/bg2597Isup3.cml

CCDC reference: 1511522

Additional supporting information: crystallographic information; 3D view; checkCIF report

## Figures and Tables

**Table 1:** Hydrogen-bond geometry (Å, °) *Cg*1 abd *Cg*2 are the centroids of the C3–C5/N1/N2 and N11/C12–C16 rings, respectively.

*D*—H⋯*A*	*D*—H	H⋯*A*	*D*⋯*A*	*D*—H⋯*A*
N5—H1⋯N11	0.88 (1)	2.00 (1)	2.7117 (7)	137 (1)
C15—H15⋯O41^i^	0.95	2.36	3.2906 (8)	165
C52—H52*B*⋯*Cg*1^ii^	0.99	2.77	3.5141 (6)	132
C53—H53*A*⋯*Cg*2^iii^	0.99	2.98	3.8761 (6)	152

Symmetry codes: (i) 
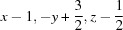
; (ii) 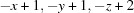; (iii) 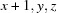.

**Table 2:** Experimental details

Crystal data	
Chemical formula	C_14_H_18_N_4_O
*M*_r_	258.32
Crystal system, space group	Monoclinic, *P*2_1_/*c*
Temperature (K)	100
*a*, *b*, *c* (Å)	9.2854 (2), 7.59144 (18), 19.4452 (5)
β (°)	102.818 (3)
*V* (Å^3^)	1336.52 (6)
*Z*	4
Radiation type	Mo *K*α
μ (mm^−1^)	0.09
Crystal size (mm)	0.10 × 0.10 × 0.05

Data collection	
Diffractometer	Rigaku MicroMax-007HF
Absorption correction	Multi-scan [*SADABS* (Bruker, 2008 ▸) and Blessing (1995 ▸)]
*T*_min_, *T*_max_	0.766, 0.996
No. of measured, independent and observed [*I* > 2σ(*I*)] reflections	14709, 6368, 5580
*R*_int_	0.016
(sin θ/λ)_max_ (Å^−1^)	0.848

Refinement	
*R*[*F* ^2^ > 2σ(*F* ^2^)], *wR*(*F* ^2^), *S*	0.037, 0.110, 1.05
No. of reflections	6368
No. of parameters	178
H-atom treatment	H atoms treated by a mixture of independent and constrained refinement
Δρ_max_, Δρ_min_ (e Å^−3^)	0.51, −0.23

Computer programs: *APEX2* and*SAINT* (Bruker, 2011 ▸), *SIR2011* (Burla *et al.*, 2012 ▸), *SHELXL2014* (Sheldrick, 2015 ▸) and *Mercury* (Macrae *et al.*, 2008 ▸).

## supplementary crystallographic information

### Crystal data

**Table:**

C_14_H_18_N_4_O	*F*(000) = 552
*M**_r_* = 258.32	*D*_x_ = 1.284 Mg m^−^^3^
Monoclinic, *P*2_1_/*c*	Mo *K*α radiation, λ = 0.71073 Å
*a* = 9.2854 (2) Å	Cell parameters from 5580 reflections
*b* = 7.59144 (18) Å	θ = 2.2–37.1°
*c* = 19.4452 (5) Å	µ = 0.09 mm^−^^1^
β = 102.818 (3)°	*T* = 100 K
*V* = 1336.52 (6) Å^3^	Block, yellow
*Z* = 4	0.10 × 0.10 × 0.05 mm

### Data collection

**Table:**

Rigaku MicroMax-007HF diffractometer	6368 independent reflections
Radiation source: Microfocus rotating anode X-ray tube, Rigaku MicroMax-007HF	5580 reflections with *I* > 2σ(*I*)
Confocal Max Flux optic monochromator	*R*_int_ = 0.016
Detector resolution: 512 pixels mm^-1^	θ_max_ = 37.1°, θ_min_ = 2.2°
Fullsphere data collection, phi and ω scans	*h* = −15→11
Absorption correction: multi-scan [SADABS (Bruker, 2008) and Blessing (1995)]	*k* = −12→9
*T*_min_ = 0.766, *T*_max_ = 0.996	*l* = −26→32
14709 measured reflections	

### Refinement

**Table:**

Refinement on *F*^2^	Primary atom site location: structure-invariant direct methods
Least-squares matrix: full	Secondary atom site location: difference Fourier map
*R*[*F*^2^ > 2σ(*F*^2^)] = 0.037	Hydrogen site location: mixed
*wR*(*F*^2^) = 0.110	H atoms treated by a mixture of independent and constrained refinement
*S* = 1.05	*w* = 1/[σ^2^(*F*_o_^2^) + (0.0645*P*)^2^ + 0.1608*P*] where *P* = (*F*_o_^2^ + 2*F*_c_^2^)/3
6368 reflections	(Δ/σ)_max_ = 0.002
178 parameters	Δρ_max_ = 0.51 e Å^−^^3^
0 restraints	Δρ_min_ = −0.23 e Å^−^^3^

### Special details

**Table:**

Experimental. It should be noted that the esd's of the cell dimensions are probably too low; they should be multiplied by a factor of 2 to 10
Geometry. All esds (except the esd in the dihedral angle between two l.s. planes) are estimated using the full covariance matrix. The cell esds are taken into account individually in the estimation of esds in distances, angles and torsion angles; correlations between esds in cell parameters are only used when they are defined by crystal symmetry. An approximate (isotropic) treatment of cell esds is used for estimating esds involving l.s. planes.

### Fractional atomic coordinates and isotropic or equivalent isotropic displacement parameters (Å^2^)

**Table:**

	*x*	*y*	*z*	*U*_iso_*/*U*_eq_	
N1	0.36539 (5)	0.84047 (6)	1.01082 (2)	0.01366 (8)	
H1	0.4865 (12)	0.6805 (13)	0.9303 (5)	0.026 (2)*	
N2	0.33841 (5)	0.91944 (6)	1.07161 (2)	0.01495 (9)	
C4	0.56919 (6)	0.79751 (7)	1.09414 (3)	0.01398 (9)	
C3	0.45948 (6)	0.89383 (7)	1.12030 (3)	0.01481 (9)	
N5	0.55256 (5)	0.68697 (7)	0.97014 (3)	0.01577 (9)	
C5	0.50458 (6)	0.76672 (7)	1.02231 (3)	0.01291 (9)	
C51	0.70133 (6)	0.62013 (7)	0.97538 (3)	0.01519 (9)	
H51A	0.7209	0.5222	1.0098	0.018*	
H51B	0.7741	0.7147	0.9921	0.018*	
C41	0.70862 (6)	0.73665 (8)	1.13548 (3)	0.01890 (11)	
H41	0.7729	0.6763	1.1117	0.023*	
O41	0.74985 (6)	0.75754 (8)	1.19936 (3)	0.02881 (12)	
C31	0.47057 (7)	0.96306 (9)	1.19315 (3)	0.02071 (11)	
H31A	0.5549	1.0435	1.2054	0.031*	
H31B	0.4843	0.8647	1.2266	0.031*	
H31C	0.3797	1.0264	1.1952	0.031*	
C16	0.16351 (7)	0.74104 (9)	0.83692 (3)	0.02033 (11)	
H16	0.1776	0.6732	0.7979	0.024*	
C15	0.03122 (7)	0.83021 (9)	0.83065 (3)	0.02048 (11)	
H15	−0.0437	0.8234	0.7886	0.025*	
C14	0.01147 (6)	0.93004 (8)	0.88782 (3)	0.01946 (11)	
H14	−0.0779	0.9931	0.8853	0.023*	
C13	0.12286 (6)	0.93706 (8)	0.94845 (3)	0.01672 (10)	
H13	0.1119	1.0050	0.9880	0.020*	
C12	0.25184 (6)	0.84098 (7)	0.94950 (3)	0.01387 (9)	
N11	0.27354 (6)	0.74497 (7)	0.89524 (3)	0.01754 (9)	
C52	0.71751 (6)	0.55527 (8)	0.90348 (3)	0.01585 (10)	
H52A	0.7075	0.6566	0.8707	0.019*	
H52B	0.6366	0.4715	0.8847	0.019*	
C53	0.86547 (6)	0.46467 (8)	0.90607 (3)	0.01637 (10)	
H53A	0.9465	0.5504	0.9214	0.020*	
H53B	0.8789	0.3679	0.9410	0.020*	
C54	0.87324 (7)	0.39047 (9)	0.83406 (3)	0.01989 (11)	
H54A	0.9708	0.3383	0.8367	0.030*	
H54B	0.8568	0.4855	0.7991	0.030*	
H54C	0.7971	0.3000	0.8202	0.030*	

### Atomic displacement parameters (Å^2^)

**Table:**

	*U*^11^	*U*^22^	*U*^33^	*U*^12^	*U*^13^	*U*^23^
N1	0.01143 (17)	0.01598 (19)	0.01227 (17)	0.00075 (14)	−0.00017 (14)	0.00018 (14)
N2	0.01383 (19)	0.01611 (19)	0.01371 (18)	0.00040 (14)	0.00052 (14)	−0.00137 (14)
C4	0.01133 (19)	0.0156 (2)	0.0135 (2)	−0.00096 (15)	−0.00037 (16)	0.00017 (16)
C3	0.0136 (2)	0.0155 (2)	0.0140 (2)	−0.00133 (16)	0.00010 (16)	−0.00071 (16)
N5	0.01264 (18)	0.0202 (2)	0.01381 (18)	0.00099 (15)	0.00157 (14)	−0.00068 (15)
C5	0.01084 (19)	0.0134 (2)	0.01358 (19)	−0.00073 (15)	0.00066 (15)	0.00134 (15)
C51	0.0126 (2)	0.0166 (2)	0.0161 (2)	−0.00029 (16)	0.00243 (16)	−0.00001 (16)
C41	0.0137 (2)	0.0238 (3)	0.0168 (2)	0.00154 (18)	−0.00179 (18)	−0.00114 (19)
O41	0.0213 (2)	0.0437 (3)	0.0168 (2)	0.0072 (2)	−0.00562 (17)	−0.00410 (19)
C31	0.0202 (2)	0.0248 (3)	0.0153 (2)	0.0004 (2)	0.00020 (19)	−0.00476 (19)
C16	0.0190 (2)	0.0280 (3)	0.0119 (2)	−0.0014 (2)	−0.00112 (18)	0.00153 (19)
C15	0.0163 (2)	0.0270 (3)	0.0152 (2)	−0.0032 (2)	−0.00273 (18)	0.00527 (19)
C14	0.0135 (2)	0.0217 (3)	0.0204 (2)	−0.00041 (18)	−0.00236 (18)	0.00482 (19)
C13	0.0127 (2)	0.0171 (2)	0.0183 (2)	0.00055 (16)	−0.00094 (17)	0.00179 (17)
C12	0.01192 (19)	0.0151 (2)	0.0130 (2)	−0.00119 (15)	−0.00053 (15)	0.00273 (15)
N11	0.0159 (2)	0.0230 (2)	0.01223 (18)	0.00039 (16)	−0.00005 (15)	0.00079 (15)
C52	0.0141 (2)	0.0184 (2)	0.0148 (2)	0.00027 (17)	0.00264 (16)	0.00057 (17)
C53	0.0143 (2)	0.0196 (2)	0.0152 (2)	0.00035 (17)	0.00317 (17)	0.00079 (17)
C54	0.0181 (2)	0.0246 (3)	0.0172 (2)	0.0018 (2)	0.00455 (19)	−0.00125 (19)

### Geometric parameters (Å, º)

**Table:**

N1—C5	1.3804 (7)	C16—N11	1.3478 (7)
N1—N2	1.3968 (7)	C16—C15	1.3841 (9)
N1—C12	1.4056 (7)	C16—H16	0.9500
N2—C3	1.3137 (7)	C15—C14	1.3909 (9)
C4—C5	1.4119 (7)	C15—H15	0.9500
C4—C3	1.4357 (8)	C14—C13	1.3862 (8)
C4—C41	1.4403 (8)	C14—H14	0.9500
C3—C31	1.4928 (8)	C13—C12	1.3986 (8)
N5—C5	1.3396 (7)	C13—H13	0.9500
N5—C51	1.4539 (7)	C12—N11	1.3340 (8)
N5—H1	0.876 (10)	C52—C53	1.5272 (8)
C51—C52	1.5208 (8)	C52—H52A	0.9900
C51—H51A	0.9900	C52—H52B	0.9900
C51—H51B	0.9900	C53—C54	1.5258 (8)
C41—O41	1.2261 (7)	C53—H53A	0.9900
C41—H41	0.9500	C53—H53B	0.9900
C31—H31A	0.9800	C54—H54A	0.9800
C31—H31B	0.9800	C54—H54B	0.9800
C31—H31C	0.9800	C54—H54C	0.9800
			
C5—N1—N2	111.99 (4)	C15—C16—H16	118.1
C5—N1—C12	129.61 (5)	C16—C15—C14	117.90 (5)
N2—N1—C12	118.37 (4)	C16—C15—H15	121.1
C3—N2—N1	105.12 (4)	C14—C15—H15	121.1
C5—C4—C3	104.82 (5)	C13—C14—C15	119.67 (6)
C5—C4—C41	129.14 (5)	C13—C14—H14	120.2
C3—C4—C41	125.89 (5)	C15—C14—H14	120.2
N2—C3—C4	112.39 (5)	C14—C13—C12	117.86 (6)
N2—C3—C31	119.97 (5)	C14—C13—H13	121.1
C4—C3—C31	127.63 (5)	C12—C13—H13	121.1
C5—N5—C51	125.11 (5)	N11—C12—C13	123.54 (5)
C5—N5—H1	114.2 (7)	N11—C12—N1	116.94 (5)
C51—N5—H1	120.7 (7)	C13—C12—N1	119.51 (5)
N5—C5—N1	121.17 (5)	C12—N11—C16	117.28 (5)
N5—C5—C4	133.17 (5)	C51—C52—C53	112.78 (5)
N1—C5—C4	105.66 (5)	C51—C52—H52A	109.0
N5—C51—C52	109.55 (4)	C53—C52—H52A	109.0
N5—C51—H51A	109.8	C51—C52—H52B	109.0
C52—C51—H51A	109.8	C53—C52—H52B	109.0
N5—C51—H51B	109.8	H52A—C52—H52B	107.8
C52—C51—H51B	109.8	C54—C53—C52	111.19 (5)
H51A—C51—H51B	108.2	C54—C53—H53A	109.4
O41—C41—C4	124.33 (6)	C52—C53—H53A	109.4
O41—C41—H41	117.8	C54—C53—H53B	109.4
C4—C41—H41	117.8	C52—C53—H53B	109.4
C3—C31—H31A	109.5	H53A—C53—H53B	108.0
C3—C31—H31B	109.5	C53—C54—H54A	109.5
H31A—C31—H31B	109.5	C53—C54—H54B	109.5
C3—C31—H31C	109.5	H54A—C54—H54B	109.5
H31A—C31—H31C	109.5	C53—C54—H54C	109.5
H31B—C31—H31C	109.5	H54A—C54—H54C	109.5
N11—C16—C15	123.75 (6)	H54B—C54—H54C	109.5
N11—C16—H16	118.1		
			
C5—N1—N2—C3	−0.35 (6)	C5—N5—C51—C52	−174.43 (5)
C12—N1—N2—C3	177.72 (5)	C5—C4—C41—O41	−173.30 (7)
N1—N2—C3—C4	−0.34 (6)	C3—C4—C41—O41	1.38 (10)
N1—N2—C3—C31	179.52 (5)	N11—C16—C15—C14	0.30 (10)
C5—C4—C3—N2	0.88 (6)	C16—C15—C14—C13	−0.09 (9)
C41—C4—C3—N2	−174.85 (5)	C15—C14—C13—C12	−0.37 (9)
C5—C4—C3—C31	−178.97 (6)	C14—C13—C12—N11	0.69 (9)
C41—C4—C3—C31	5.29 (10)	C14—C13—C12—N1	−178.05 (5)
C51—N5—C5—N1	174.02 (5)	C5—N1—C12—N11	6.28 (8)
C51—N5—C5—C4	−5.74 (10)	N2—N1—C12—N11	−171.40 (5)
N2—N1—C5—N5	−178.93 (5)	C5—N1—C12—C13	−174.90 (5)
C12—N1—C5—N5	3.28 (9)	N2—N1—C12—C13	7.42 (7)
N2—N1—C5—C4	0.89 (6)	C13—C12—N11—C16	−0.49 (9)
C12—N1—C5—C4	−176.91 (5)	N1—C12—N11—C16	178.28 (5)
C3—C4—C5—N5	178.77 (6)	C15—C16—N11—C12	−0.02 (9)
C41—C4—C5—N5	−5.69 (10)	N5—C51—C52—C53	−173.79 (5)
C3—C4—C5—N1	−1.02 (6)	C51—C52—C53—C54	176.10 (5)
C41—C4—C5—N1	174.53 (6)		

### Hydrogen-bond geometry (Å, º)

Cg1 abd Cg2 are the centroids of the C3–C5/N1/N2 and N11/C12–C16 rings, respectively.

**Table:**

*D*—H···*A*	*D*—H	H···*A*	*D*···*A*	*D*—H···*A*
N5—H1···N11	0.876 (10)	2.004 (11)	2.7117 (7)	137.0 (9)
C15—H15···O41^i^	0.95	2.36	3.2906 (8)	165
C52—H52*B*···*Cg*1^ii^	0.99	2.77	3.5141 (6)	132
C53—H53*A*···*Cg*2^iii^	0.99	2.98	3.8761 (6)	152

Symmetry codes: (i) *x*−1, −*y*+3/2, *z*−1/2; (ii) −*x*+1, −*y*+1, −*z*+2; (iii) *x*+1, *y*, *z*.
